# Sequential Cohort Design Applying Propensity Score Matching to Analyze the Comparative Effectiveness of Atorvastatin and Simvastatin in Preventing Cardiovascular Events

**DOI:** 10.1371/journal.pone.0090325

**Published:** 2014-03-10

**Authors:** Arja Helin-Salmivaara, Piia Lavikainen, Emma Aarnio, Risto Huupponen, Maarit Jaana Korhonen

**Affiliations:** 1 Department of Pharmacology, Drug Development and Therapeutics, University of Turku, Turku, Finland; 2 Unit of Primary Health Care, Hospital District of Helsinki and Uusimaa, Helsinki, Finland; 3 Department of Clinical Pharmacology, Tykslab, Turku University Hospital, Turku, Finland; 4 School of Pharmacy, University of Eastern Finland, Kuopio, Finland; 5 Department of Public Health, University of Turku, Turku, Finland; Emory University, United States of America

## Abstract

**Background:**

Sequential cohort design (SCD) applying matching for propensity scores (PS) in accrual periods has been proposed to mitigate bias caused by channeling when calendar time is a proxy for strong confounders. We studied the channeling of patients according to atorvastatin and simvastatin initiation in Finland, starting from the market introduction of atorvastatin in 1998, and explored the SCD PS approach to analyzing the comparative effectiveness of atorvastatin versus simvastatin in the prevention of cardiovascular events (CVE).

**Methods:**

Initiators of atorvastatin or simvastatin use in the 45–75-year age range in 1998–2006 were characterized by their propensity of receiving atorvastatin over simvastatin, as estimated for 17 six-month periods. Atorvastatin (10 mg) and simvastatin (20 mg) initiators were matched 1∶1 on the PS, as estimated for the whole cohort and within each period. Cox regression models were fitted conventionally, and also for the PS matched cohort and the periodically PS matched cohort, to estimate the hazard ratios (HR) for CVEs.

**Findings:**

Atorvastatin (10 mg) was associated with a 11%–12% lower incidence of CVE in comparison with simvastatin (20 mg). The HR estimates were the same for a conventional Cox model (0.88, 95% confidence interval 0.85–0.91), for the analysis in which the PS was used to match across all periods and the Cox model was adjusted for strong confounders (0.89, 0.85–0.92), and for the analysis in which PS matching was applied within sequential periods (0.88, 0.84–0.92). The HR from a traditional PS matched analysis was 0.80 (0.77–0.83).

**Conclusions:**

The SCD PS approach produced effect estimates similar to those obtained in matching for PS within the whole cohort and adjusting the outcome model for strong confounders, but at the cost of efficiency. A traditional PS matched analysis without further adjustment in the outcome model produced estimates further away from unity.

## Introduction

Comparative effectiveness analyses of pharmaceuticals in observational settings are prone to bias due to confounding and channeling. Channeling, the preferential prescribing of one drug over another for various reasons, may lead to confounding when selective prescribing is based on patient characteristics associated with the outcome of interest. When changes in channeling occur over time, calendar time itself is a potential confounder or is a proxy for other confounders [1], [2]. Therefore, calendar time is a key component when the effects of a newly launched drug are compared with those of a pre-existing one. Sequential cohort design (SCD) applying propensity score (PS) matching has been proposed as a means of mitigating bias caused by channeling when calendar time is a proxy for strong confounders [1]–[3]. In this approach, PSs for receiving one treatment over another are first constructed for cohorts within a selected number of sequential periods. For each PS model, the effects of covariates on treatment selection may vary, and even different sets of covariates can be used according to the time and the availability of the covariates [1], [2]. The persons in the comparison cohorts are then matched on PS. Matching according to PSs within study-specific periods increases the covariate balance [4] and hence enhances the comparability of the cohorts. Thereafter, analyses on exposure-outcome associations are conducted using period as a stratum.

Seeger at al. explored a similar design in comparing the incidence of myocardial infarction between initiators of statin therapy and non-initiators in a US health plan in the 1990's [2]. More recently, the design has been applied in comparative effectiveness studies on cancer chemotherapy [3] and second-generation antipsychotics [4], as well as in simulation studies on drug safety monitoring of drug therapies [5], [6]. Applications of the design in settings outside US are scarce however.

In a previous study [7], we demonstrated that, during the first 4 years after its introduction into the Finnish market in 1998, atorvastatin was channeled to younger and healthier sectors of the population than simvastatin (introduced in the early 1990's) was. By 2004, however, the differences between atorvastatin and simvastatin initiators in the distributions of age and the number of cardiovascular drugs in use had disappeared [7]. In our present study, we have described the channeling of atorvastatin over simvastatin overall in 1998–2006 in Finland. We explored application of the SCD approach to analyzing the comparative effectiveness of atorvastatin versus simvastatin in the prevention of cardiovascular events (CVE) among new statin users. We assumed that, when used in equipotent doses, atorvastatin and simvastatin would be equally effective in preventing CVEs (i.e., the effect estimate would approach unity). Furthermore, by iterating the survival analysis stratified by the cohort accrual periods, we simulated accumulating data by time. The data were captured from nationwide health care registers.

## Methods

### Sources of Data

We used data from administrative health databases generated in Finland through the universal health care and drug reimbursement systems covering the 5.3 million residents. We identified prescription records since 1994 in the Prescription Register, which is managed by the Social Insurance Institution of Finland (SII) [8]. This register contains records of all reimbursed prescription drug purchases made by residents in non-institutional settings. For each purchase, the dispensing date, the Anatomical Therapeutic Chemical classification code of the WHO [9], the tablet strength, and the quantity dispensed are listed. Patients staying in a public nursing home or hospital without interruption for over 90 days are not eligible for drug reimbursement, and their purchases are not registered. We identified these patients from a separate SII register. For identifying patients entitled to higher rates of reimbursement because of certain severe, chronic conditions, we used the SII Special Reimbursement Register introduced in 1964. To be eligible for special reimbursement, a patient's condition must meet explicit predefined criteria, and a written certificate by a specialist physician is required.

We identified hospitalizations from the Finnish Care Register, managed by the National Institute for Health and Welfare. The register, covering all Finnish hospitals, includes individual-level administrative data on main and additional discharge diagnoses, as well as the admission and discharge dates. The 10th revision of the International Classification of Diseases (ICD-10) has been in use since 1 January 1996. The data from the databases were linked anonymously using encrypted personal identifiers.

### Analyses

#### Synopsis

All initiators of atorvastatin or simvastatin use in 1998–2006 were characterized by the distributions of the covariates measured at baseline and the PSs for receiving atorvastatin over simvastatin estimated for each of 17 six-month periods. After restricting the cohort to those initiating atorvastatin (10 mg) or simvastatin (20 mg) use and with follow-up of at least 270 days since the initiation, we estimated the PSs for the entire period (1998–2006) and also for the 17 six-month periods. The atorvastatin and simvastatin initiators were matched 1∶1 on the PS. Cox regression models were fitted both conventionally and for the PS matched cohorts to estimate the hazard ratios for CVEs occurring during the follow-up until 31 December 2008. Finally, hazard ratios were estimated cumulatively (simulating accruing data in real life) with a sequentially PS matched analysis and with conventional (unmatched) multivariable analyses. In these analyses, the follow-up was restricted to 730 days.

#### Cohorts

The initiators of statin therapy with simvastatin or atorvastatin between January 1998 and June 2006 and in the age range of 45–75 years were drawn from the SII Prescription Register. The initiation was defined as not having purchased any statin between 1 January 1994 and the date of the first simvastatin or atorvastatin purchase, which was set as the index date. Starting from the first half of 1998, the initiators were categorized into 17 cohorts according to the 6-month period of their index date. We estimated separate PSs for each period with a logistic regression by modeling the predicted probability of receiving atorvastatin as a function of covariates. The following covariates were used: demographic characteristics, number of hospital days in the preceding 365 days, prior cardiovascular disease in the preceding 7 years, comorbidities and medication used in the preceding 365 days, and the number of distinct drugs purchased during the 4 months prior to the initiation. A detailed list of the covariates, other than place of residence (categorized into 21 catchment areas of the secondary/tertiary care hospitals), is presented in Table 1. The covariates included in each PS model varied and reflected the time and changes in the availability of the covariates. The PS distributions of atorvastatin and simvastatin were compared within each period.

**Table 1:** Characteristics of the initiators of simvastatin and atorvastatin therapy between January 1998 and June 2006 in Finland.

	Initiators without matching			Sequentially PS matched		
	Simvastatin 20 mg	Atorvastatin 10 mg		Simvastatin 20 mg	Atorvastatin 10 mg	
	(*n* = 73 868)	(*n* = 96 995)		(*n* = 54 220)	(*n* = 54 220)	
	*n* (%)	*n* %	*P* value	*n* (%)	*n* %	*P* value
Female	36 612 (49.6)	50 107 (51.7)	<0.001	27 453 (50.6)	27 449 (50.6)	*
Age, mean (SD)	60.9 (8.0)	60.4 (8.0)	<0.001	60.7 (8.0)	60.7 (8.0)	*
**Age category**			<0.001			*
45–55 years	21 200 (28.7)	30 431 (31.4)		15 992 (29.5)	15 868 (29.3)	
56–65 years	29 252 (39.6)	37 674 (38.8)		21 400 (39.5)	21 338 (39.4)	
66–75 years	23 416 (31.7)	28 890 (29.8)		16 828 (31.0)	17 014 (31.4)	
**Number of hospital days during 365 days prior to the initiation**			<0.001			*
0	51 583 (69.8)	73 396 (75.7)		39 233 (72.4)	39 265 (72.4)	
1–7	14 202 (19.2)	15 908 (16.4)		9837 (18.1)	9835 (18.1)	
8–30	6790 (9.2)	6281 (6.5)		4262 (7.9)	4204 (7.8)	
31–365	1293 (1.8)	1410 (1.5)		888 (1.6)	916 (1.7)	
**Comorbidities**						
*CVD, PTCA or CAPG in relation to the initiation*			<0.001			*
None in preceding 7 years	63 469 (85.9)	82 625 (91.4)		48 130 (88.8)	48 166 (88.8)	
Only earlier than 30 days	3700 (5.0)	4791 (4.9)		2846 (5.3)	2833 (5.2)	
Only during the preceding 30 days	5743 (7.8)	2916 (3.0)		2705 (5.0)	2680 (4.9)	
Both prior to and during 30 days	956 (1.3)	663 (0.7)		539 (1.0)	541 (1.0)	
*Hospitalized during 7 years prior to the initiation*						
Diabetes	3589 (4.9)	4144 (4.3)	<0.001	2471 (4.6)	2493 (4.6)	*
Hypertension	5269 (7.1)	5933 (6.1)	<0.001	3593 (6.6)	3620 (6.7)	*
Stroke	3692 (5.0)	5755 (5.9)	<0.001	2972 (5.5)	2838 (5.2)	0.071
Cardiac insufficiency	990 (1.3)	814 (0.8)	<0.001	594 (1.1)	567 (1.1)	*
Atherosclerotic CVD	988 (1.3)	1195 (1.2)	0.054	710 (1.3)	711 (1.3)	*
Atherosclerosis in lower legs	416 (0.6)	507 (0.5)	0.258	295 (0.5)	288 (0.5)	*
	(*n* = 73 868)	(*n* = 96 995)		(*n* = 54 220)	(*n* = 54 220)	
	*n* (%)	*n* %	*P* value	*n* (%)	*n* %	*P* value
*Hospitalized during 365 days prior to the initiation*						
Atrial fibrillation	957 (1.3)	986 (1.0)	<0.001	655 (1.2)	614 (1.1)	*
Any cancer diagnosis	461 (0.6)	578 (0.6)	0.458	316 (0.6)	315 (0.6)	*
COPD/asthma	324 (0.4)	291 (0.3)	<0.001	207 (0.4)	207 (0.4)	*
Renal insufficiency	22 (0.0)	50 (0.1)	0.030	12 (0.0)	21 (0.0)	*
Dementia	36 (0.1)	35 (0.0)	0.204	19 (0.0)	21 (0.0)	*
Psychotic disease	180 (0.2)	206 (0.2)	0.177	127 (0.2)	123 (0.2)	*
Depression	189 (0.3)	202 (0.2)	0.041	124 (0.2)	126 (0.2)	*
Organ transplantation	8 (0.0)	13 (0.0)	0.634	3 (0.0)	4 (0.0)	*
*Comorbidities based on the Special Reimbursement Register* #						
Diabetes	8161 (11.1)	10 043 (10.4)	<0.001	5703 (10.5)	5760 (10.6)	*
Hypothyroidism	2494 (3.4)	3295 (3.4)	0.814	1866 (3.4)	1857 (3.4)	*
Psychotic disorders	2015 (2.7)	2319 (2.4)	<0.001	1345 (2.5)	1350 (2.5)	*
Severe psychotic disease	147 (0.2)	137 (0.1)	<0.001	96 (0.2)	97 (0.2)	*
Breast cancer	361 (1.0)	427 (0.9)	0.150	256 (1.0)	257 (1.0)	*
Prostate cancer	364 (0.9)	387 (0.9)	<0.001	243 (0.9)	242 (0.9)	*
Leukemia	227 (0.3)	229 (0.2)	0.005	145 (0.3)	140 (0.3)	*
Gynecologic cancers	42 (0.1)	68 (0.1)	0.285	34 (0.1)	27 (0.1)	*
Other cancers	85 (0.1)	89 (0.1)	0.135	45 (0.1)	48 (0.1)	*
Prior organ transplantation	69 (0.1)	139 (0.1)	0.003	56 (0.1)	52 (0.1)	*
Uremia with dialysis	40 (0.1)	104 (0.1)	<0.001	28 (0.0)	33 (0.1)	*
Use of interferon alpha	13 (0.0)	19 (0.0)	0.766	8 (0.0)	9 (0.0)	*
Cardiac insufficiency	2001 (2.7)	2181 (2.3)	<0.001	1341 (2.5)	1350 (2.5)	*
Rheumatic disease	2117 (2.9)	2559 (2.6)	<0.001	1492 (2.8)	1480 (2.7)	*
Asthma	5194 (7.0)	5867 (6.1)	<0.001	3576 (6.6)	3592 (6.6)	*
Chronic hypertension	22 723 (30.8)	30 214 (31.1)	0.085	16 776 (30.9)	16 819 (31.0)	*
	(*n* = 73 868)	(*n* = 96 995)		(*n* = 54 220)	(*n* = 54 220)	
	*n* (%)	*n* %	*P* value	*n* (%)	*n* %	*P* value
CAD	10 917 (14.9)	10 434 (10.8)	<0.001	7049 (13.0)	7034 (13.0)	*
Dysrhythmia	1707 (2.3)	2037 (2.1)	<0.001	1231 (2.3)	1223 (2.3)	*
Familial dyslipidemia	40 (0.1)	36 (0.0)	0.098	26 (0.1)	25 (0.1)	*
Dyslipidemia with CAD	3692 (5.0)	2927 (3.0)	<0.001	2256 (4.2)	2256 (4.2)	*
Clopidogrel use with CAD	74 (0.1)	43 (0.0)	<0.001	36 (0.1)	36 (0.1)	*
Clopidogrel use with other indications	235 (0.3)	220 (0.2)	<0.001	146 (0.3)	149 (0.3)	*
Use of anti-dementia drugs§	128 (0.2)	206 (0.2)	0.070	96 (0.2)	91 (0.2)	*
Parkinsonism	241 (0.3)	274 (0.3)	0.102	163 (0.3)	145 (0.3)	*
Epilepsy	847 (1.2)	1165 (1.2)	0.301	621 (1.2)	614 (1.1)	*
**Medication**						
*Number* of different preparations purchased during 4 months prior to the initiation			<0.001			
1–5	9571 (13.0)	7626 (7.9)		5675 (10.5)	5749 (10.6)	*
6–10	20 434 (27.7)	21 861 (22.5)		14 015 (25.9)	14 034 (25.9)	
11–15	17 596 (23.8)	22 806 (23.5)		13 108 (24.2)	13 087 (24.1)	
16–20	11 339 (15.4)	17 041 (17.6)		8828 (16.3)	8884 (16.4)	
>20	14 928 (20.2)	27 661 (28.5)		12 594 (23.2)	12 466 (23.0)	
*At least one purchase during 365 days prior to the initiation*						
Diabetes drugs	11 500 (15.6)	14 284 (14.7)	<0.001	8026 (14.8)	8027 (14.8)	*
Antithrombotic agents	8608 (11.7)	9850 (10.2)	<0.001	5887 (10.9)	5814 (10.7)	*
Organic nitrates and cardiac glycosides	15 772 (21.4)	15 877 (16.4)	<0.001	10 385 (19.2)	10 309 (19.0)	*
Centrally acting hypertension drugs	644 (0.9)	746 (0.8)	0.019	437 (0.8)	441 (0.8)	*
Diuretics	11 203 (15.2)	14 231 (14.7)	<0.001	8066 (14.9)	8050 (14.9)	*
Peripheral vasodilators	64 (0.1)	124 (0.1)	0.011	49 (0.1)	52 (0.1)	*
Beta-blocking agents	31 409 (42.5)	36 108 (37.2)	<0.001	21 906 (40.4)	21 915 (40.4)	*
Selective calcium channels blockers	12 678 (17.2)	16 133 (16.6)	<0.001	9203 (17.0)	9270 (17.1)	*
ACEI or ARB	26 292 (36.0)	30 162 (31.1)	<0.001	18 166 (33.5)	18 176 (33.5)	*
	(*n* = 73 868)	(*n* = 96 995)		(*n* = 54 220)	(*n* = 54 220)	
	*n* (%)	*n* %	*P* value	*n* (%)	*n* %	*P* value
Non-statin lipid-lowering drug	544 (0.7)	1573 (1.6)	<0.001	439 (0.8)	472 (0.9)	*
Drugs for obstructive airway diseases	7680 (10.4)	9293 (9.6)	<0.001	5361 (9.9)	5387 (9.9)	*
Anti-dementia drugs	79 (0.1)	111 (0.1)	0.645	59 (0.1)	54 (0.1)	*
Antidepressants	7086 (9.6)	9424 (9.7)	0.393	5232 (9.7)	5172 (9.5)	*
Antipsychotics	2088 (2.8)	2555 (2.6)	0.015	1430 (2.6)	1437 (2.7)	*
Antineoplastic agents	342 (0.5)	521 (0.6)	0.032	265 (0.5)	256 (0.5)	*

The study population restricted by the strength of the initiating drug and by the start of the follow-up at 270 days since the initiation is presented without and with the sequential matching by propensity score.

CVD, cardiovascular disease; PTCA, percutaneous transluminal coronary angioplasty; CAPG, coronary artery bypass craft surgery; COPD, chronic obstructive pulmonary disease; CAD, coronary artery disease; ACEI, angiotensin–converting-enzyme inhibitor; ABR, angiotensin receptor blocker.

# Eligibility to special reimbursement any time prior to the initiation.

§ Use of donepezil, galantamine, memantine, or rivastigmine.

*P* values are based on Chi^2^ test for categorical variables and Student's *t* test for continuous variables.

* >0.1.

For the effectiveness analyses, the study population was restricted to the initiators with atorvastatin (10 mg) or simvastatin (20 mg). Atorvastatin in 10 mg doses has been reported to equal the potency of simvastatin in 20 mg doses [10] and simvastatin in 40 mg doses [11] in lowering the levels of low-density lipoprotein. We further restricted the population to those with a follow-up starting on the 270th day to avoid potential protopathic bias [12].

#### PS matching

After the restrictions were made, we estimated the PSs for the whole period (1998–2006) and for the 17 six-month periods by using the covariates. Within each period, an initiator with atorvastatin was matched to an initiator with simvastatin within a 0.01 caliper of propensity score, and the initiators without counterparts were excluded [13]. The balance of the key covariates was tested by calculating standardized mean differences [14]. The covariate distributions were displayed for the pooled cohorts.

#### Comparative effectiveness analyses

A survival analysis of time from the 270^th^ day since the initiation or matching to CVE was estimated using Cox proportional hazard regression applying the intent-to-treat approach. The follow-up ended in death, institutionalization, 31 December 2008, or the outcome of interest, whichever came first. The primary outcome was a hospitalized CVE, a composite of acute myocardial infarction, ischemic cardiac disease (ICD codes I20.0, I20.1, I20.8, I20.9, I21.0–I21.9, I22.0, I22.1, I22.8, I22.9, I23.0–I23.5, I23.8, I24.0, I24.1, I24.8, I24.9), percutaneous coronary intervention or coronary artery bypass surgery, and ischemic stroke (I63, I64). Both the main and additional diagnoses were selected. The validity of the measures in the Care Register has been reported to be fairly good in that, when myocardial infarction and unstable angina pectoris diagnoses combined were compared with the population-based FINAMI register, the positive predictive value was 76% for males and 69% for females in 1998–2002 [15]. The positive predictive value of the first stroke diagnosis in the Care Register compared with the population-based FINSTROKE register was 85% for 1996–2002 [16].

Hazard ratios were estimated with Cox models using the following three different PS approaches: 1) a PS estimated across all periods combined (period included in the logistic regression model for PS) and used for matching, 2) a PS estimated across all periods combined and used for matching as above and the outcome model adjusted for variables strongly predicting the outcome (p<0.001), and 3) a PS estimated and used for matching within the cohort accrual periods. Furthermore, conventional outcome models using the same covariates as in the logistic model for the PS were fitted.

#### Cumulative Analyses

To simulate real life, we added the subsequent matched cohort to the previous ones and iterated the survival analysis stratified by the cohort accrual periods 16 times. For the unmatched accrual cohorts, Cox proportional regression models were adjusted for the same covariates as included in the PSs. In these analyses, the follow-up was restricted to between the 270^th^ and 730^th^ days since the initiation.

We used SAS software (version 9.2, SAS Institute, Inc., Cary, NC, USA) for the statistical analyses.

#### Ethics Statement

Data were obtained from the databases hosted by the SII and the National Institute for Health and Welfare, Helsinki, Finland that are not public repositories. The SII, the National Institute for Health and Welfare, and the national data protection agency (Office of the Data Protection Ombudsman) approved the study protocol.

There was no legal requirement for an ethics committee approval because researchers used only de-identified register data and the persons in the registers were not contacted (the Finnish legislation at: http://www.finlex.fi/fi/laki/ajantasa/1999/19990488 - not available in English). No written consent from patients was required either. Data were de-identified by the SII after the record linkage. De-identified data can be shared by permission only.

## Results

### All Initiators

**Figure 1:**
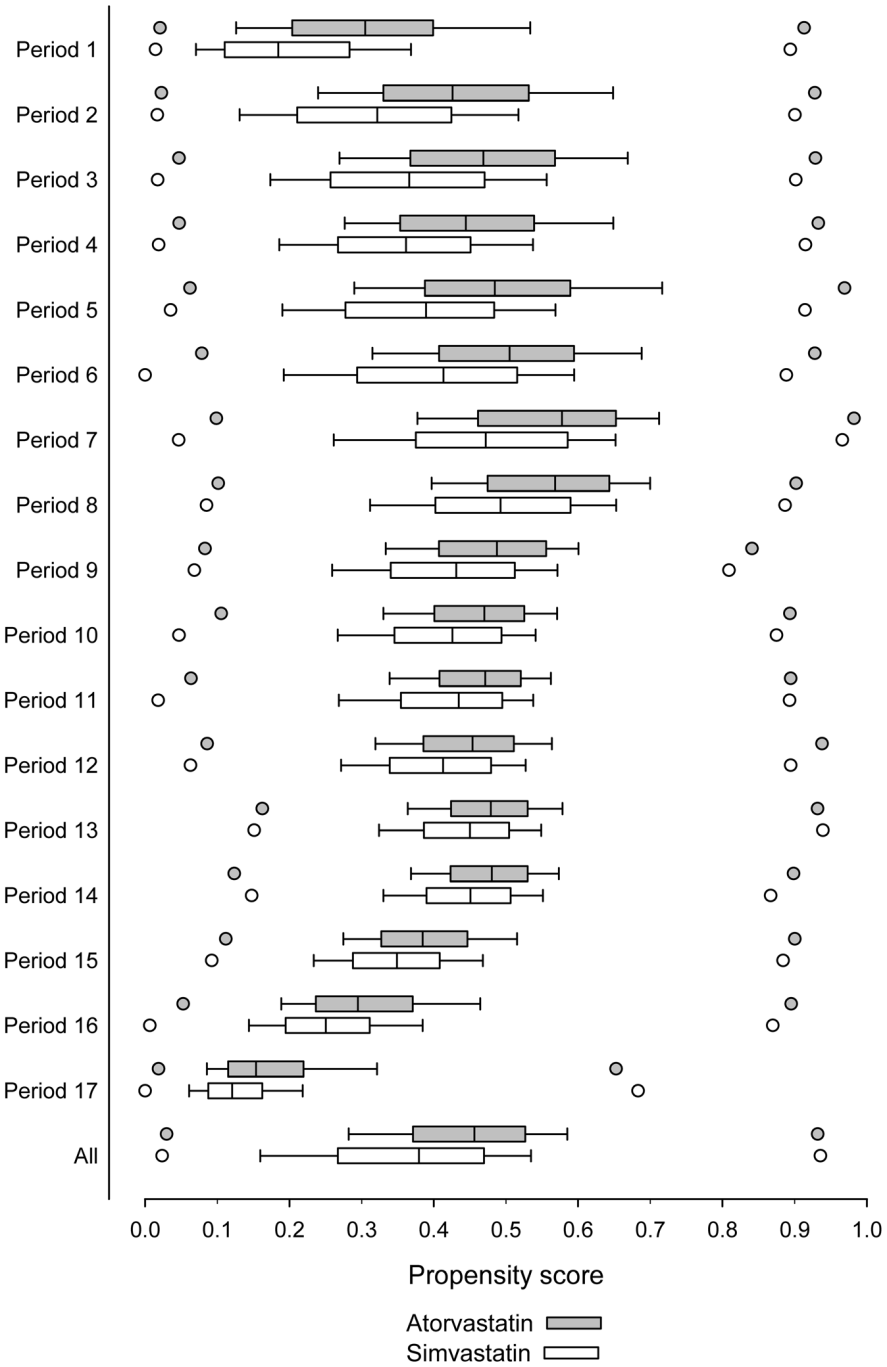
Distribution of propensity scores estimating the effect of initiating statin therapy with simvastatin versus atorvastatin in 1998–2006 in 6-month periods (1^st^ = from 1 January 1998 to 30 June 1998; 17^th^ = from 1 January 2006 to 30 June 2006). The boundary of the box closest to zero indicates the 25th percentile, a line within the box marks the median, and the boundary of the box farthest from zero indicates the 75th percentile. Whiskers (error bars) above and below the box indicate the 90th and 10th percentiles. In addition, the lowest and the highest values are presented by points.

Between January 1998 and June 2006 in Finland, 118 623 persons initiated atorvastatin use and 180 238 began simvastatin therapy. The mean age of the atorvastatin initiators was 60.3 (SD 8.0) years and that of simvastatin initiators was 61.4 (SD 8.0) years (Table S1). The initiators of atorvastatin tended to have slightly fewer comorbid conditions than their comparison group when the persons in all of the periods were pooled (Table S1). Over the periods, however, the prevalence of prior CVD identified in the discharge register changed remarkably. In the first half of 1998, 80.8% of the initiators with atorvastatin and 67.7% with simvastatin did not have prior CVD. In the first half of 2006, the respective proportions were 83.3% and 90.97%. As shown in Figure 1, the medians of the PSs for the atorvastatin and simvastatin initiators were the closest in period 11 (the first half of 2003) (i.e., 0.47 and 0.44, respectively). The overlap of the distributions started increasing in the first half of 2001.

### Restrictions and Matching

After restrictions according to the strength of the initiating statin and the start of the follow-up, the atorvastatin cohort included 96 995 persons, and the simvastatin cohort had 73 868 persons. The mean age of the atorvastatin initiators was 60.4 (SD 8.0) years, and that of the simvastatin initiators was 60.9 (SD 8.0) (Table 1). As in the cohort containing all of the initiators, the persons with atorvastatin tended to have slightly fewer comorbid conditions. After the PS matching across all of the cohorts combined, 128 540 persons from both groups retained; 66.2% of the restricted atorvastatin cohort and 87% of the respective simvastatin cohort. After sequential PS matching, 54 220 persons were retained in each group (55.9% of the restricted atorvastatin cohort and 73.4% of the respective simvastatin cohort, the proportions varying across the periods) (Table S2). The covariate balance increased after the restrictions and PS matching (Table 1, Table S3).

### Comparative Effectiveness Analyses

During the 256 060 person-years of the atorvastatin (10 mg) initiators followed-up since the 270^th^ day after the initiation until censoring or experiencing an event, 3795 CVEs were observed, yielding a crude incidence of 15/1000 person-years. For the simvastatin (20 mg) initiators, 4261 CVEs in 253 278 person-years yielded an incidence of 17/1000 person-years. The larger person-time of the atorvastatin initiators was mainly due to the larger number of deaths among simvastatin initiators (4.01% versus 4.26%).

Atorvastatin (10 mg) was associated with an 11%–12% lower incidence of CVE in comparison with simvastatin (20 mg). The hazard ratio (HR) estimated with a conventionally adjusted Cox model was the same as the one obtained in the analysis in which the PS was used for matching across all periods and the Cox model was adjusted for strong predictors of the outcome, as well as the one from the analysis in which the PS matching was applied within sequential periods: 0.88, 0.89, and 0.88 with nearly identical confidence intervals (CI) (95% CI 0.85–0.91, 0.85–0.92, and 0.84–0.92, respectively) (Table 2). However, the HR from the analysis in which PS matching was used across all of the periods without further adjustment of the outcome model for confounders produced an HR of 0.80 (95% CI 0.77–0.83).

**Table 2:** Comparative effectiveness of atorvastatin (10 mg) versus simvastatin (20 mg).

Models	HR (95% CI)
**Conventional outcome models**	
Unadjusted (*n* = 170 763)	0.70 (0.68–0.73)
Adjusted (*n* = 170 763)^2^	0.88 (0.85–0.91)
**PS models**	
Matched by PS, unadjusted (*n* = 128 540)^3^	0.80 (0.77–0.83)
Matched by PS, adjusted (*n* = 128 540)^4^	0.89 (0.85–0.92)
Sequentially matched by PS (*n* = 108 440)^5^	0.88 (0.84–0.92)

The analyses included initiators between January 1998 and June 2006 in Finland. Hazard ratios for a composite of cardiovascular events^1^ were estimated with different Cox proportional hazard regression models.

HR = hazard ratio; CI = confidence interval; PS = propensity score.

1 Cardiovascular events; hospitalized acute myocardial infarction ischemic cardiac disease (ICD-10 codes I20–I24), percutaneous coronary intervention or coronary artery bypass surgery, and stroke (I63, I64).

2 Adjusted for all covariates included in the propensity scores, including the period.

3 Period included in the propensity score. Matching within the whole cohort within a 0.01 caliber.

4 Adjusted for the covariates strongly associated with the outcome.

5 Matching conducted within each of the 17 six-month periods since January 1998.

### Cumulative Analyses

For each period, the HRs estimated with conventional models tended to be slightly closer to one than the HRs from the models applying PS matching, with the exception of the first two periods (Table 3).

**Table 3:** Comparative effectiveness of atorvastatin (10 mg) versus simvastatin (20 mg) estimated cumulatively.

	Sequentially matched by PS^2^	Conventional^3^
Period	HR (95% CI)	HR (95% CI)
1	0.88 (0.54–1.43)	0.85 (0.56–1.30)
2	1.02 (0.75–1.40)	1.11 (0.85–1.44)
3	0.89 (0.70–1.12)	0.89 (0.73–1.09)
4	0.85 (0.70–1.03)	0.88 (0.75–1.04)
5	0.83 (0.71–0.98)	0.83 (0.72–0.96)
6	0.76 (0.66–0.88)	0.77 (0.68–0.88)
7	0.77 (0.68–0.88)	0.78 (0.70–0.88)
8	0.78 (0.69–0.88)	0.80 (0.72–0.89)
9	0.78 (0.70–0.87)	0.80 (0.72–0.88)
10	0.80 (0.72–0.89)	0.82 (0.75–0.90)
11	0.80 (0.72–0.88)	0.81 (0.75–0.89)
12	0.81 (0.73–0.89)	0.82 (0.75–0.89)
13	0.83 (0.75–0.90)	0.83 (0.77–0.90)
14	0.83 (0.76–0.91)	0.84 (0.78–0.90)
15	0.86 (0.79–0.93)	0.86 (0.80–0.92)
16	0.87 (0.80–0.94)	0.87 (0.81–0.93)
17	0.86 (0.80–0.93)	0.87 (0.81–0.93)

The analyses included the initiators between January 1998 and June 2006 in Finland; hazard ratios of a composite of cardiovascular events^1^ estimated cumulatively in the sequentially matched cohorts and in conventional Cox proportional hazard regression models. The follow-up was restricted to between 270 and 730 days since initiation.

HR = hazard ratio; CI = confidence interval; PS = propensity score.

1 Cardiovascular events: hospitalized acute myocardial infarction (ICD-10 codes I20–I24), percutaneous coronary intervention or coronary artery bypass surgery, and stroke (I63, I64).

2 Matching conducted within each of the 17 six-month periods since January 1998.

3 Adjusted for all of the covariates included in the propensity scores, including the period.

## Discussion

The difference in the characteristics of the atorvastatin and simvastatin initiators overall was shown by the distribution of the PSs, a sum function of the covariates measured at treatment initiation. According to the PSs, the covariates were extremely well balanced after sequential matching in the cohorts restricted by the strength of the initiating statin and by the start of the follow-up. Our comparative effectiveness analysis indicated that atorvastatin (10 mg) was more effective than simvastatin (20 mg) for the prevention of CVEs. The HRs estimated with the Cox proportional hazard models were the same whether derived from the sequential cohort approach or the conventional model. No substantial difference in the effect estimates was found between the sequential cohort and conventional approaches when the accumulation of data was extrapolated to real life and the follow-up was restricted to 2 years.

### Channeling

As PS is a relative sum function, the interpretation of our findings is challenging. Since the first half of 2001 (period 7), the interquartile range of the PS distributions increasingly overlapped, although a common trend for the PSs can be found. The distribution of the PSs was visually classified into the following three phases; the phase of increasing scores from 1998 to 2001, a stable phase between 2002 and 2004, and a phase of decreasing scores since 2005 (Figure 1). The start of the plateau phase coincides with the withdrawal of cerivastatin from the market [17]. The withdrawal hardly directly affects the preferential prescribing of either statin or, consequently, the PSs. The absolute number of initiators of both statins, however, decreased during the period following the withdrawal (period 8 in Table S2). This decrease possibly reflects mistrust towards the safety of statin drugs. The end of the plateau phase coincided with a substantial decrease in the prices of the generic simvastatin products [18]. Generic substitution was launched in Finland in April 2003. We assume that the price gradient between the branded atorvastatin and generic simvastatin (and other generic statins) was great enough to affect prescribing practices as late as 2005. During the last three periods (Table S2), initiations clearly shifted toward simvastatin products. A policy change restricting reimbursement for atorvastatin was implemented in October 2006; however, the fact that this change was forthcoming was not made known until the preceding June.

### Comparative Effectiveness

The effect estimate did not reach unity in any of our comparative effectiveness analyses. The strengths of atorvastatin (10 mg) and simvastatin (20 mg) may not compare equally. For feasible analyses we had, however, too few of those initiating simvastatin (40 mg) use in the first years of the study. In a systematic review [11], the percentage of reduction in serum low-density lipoprotein by simvastatin (20 mg) was 32% and that for atorvastatin (10 mg) was 37%, yielding a potency ratio of 0.86, near the HRs in our exploratory comparative effectiveness analyses.

We compared, however, the effectiveness of atorvastatin (10 mg) versus simvastatin (40 mg) in the whole cohort of initiators; that is, we estimated a PS across all periods combined and used the PS for matching within the whole cohort. In this analysis, HRs approached the unity (HR 0.94; 95% CI 0.84–1.06) (Table S4).

As the analyses were all intention-to-treat, neither switching between statins, changes in the doses, nor discontinuation of, or adherence to statin therapy was accounted for. Therefore, a misclassification of person-time was possible.

Head-to-head comparisons of the effects of atorvastatin versus simvastatin on cardiovascular outcomes are scarce. In the only large enough comparative randomized trial, 75% of the participants had pre-randomization statin therapy, which diluted the possible difference [19]. In several observational studies conducted among privately insured or employer insured populations in the US at the beginning of the 2000's, the initiators of atorvastatin (10/20 mg) use tended to have a lower risk of cardiovascular outcomes than those initiating simvastatin (20/40 mg] use during a few years of follow-up [20]–[23]. In the conventionally adjusted predictive models, the HRs ranged from 0.87 [21] to 0.98 [22]. In a study in which PS matching was applied to the whole cohort, the relative risk was 0.91 (p = 0.02) [23].

### Analyses of Comparative Effectiveness

For valid effect estimates, correct specification of the PS model is essential. If the associations between the covariates and the exposure substantially differ across subgroups, applying cohort-wide PS in subgroup analyses may lead to biased estimates. In our exploration, the effect estimates from the cohort-wide PS analysis improved (i.e., approached unity) when we added strong predictors of the outcome (i.e., age, sex, prior CVD, drugs used for the modification of CVD risks, number of hospital days, and number of different drugs redeemed) to the outcome model (Table 2). Actually, the estimate became identical to the one from the sequential cohort model. Correct matching for PS, on the other hand, can be estimated by covariate balance. In our study, the balance of the key patient characteristics predicting CVEs, calculated as the standardized mean difference, improved after cohort-wide PS matching when compared with that of the unmatched study population. The balance was, however, further improved after matching within periods (Table S3). It must be noted that, due to the limitations of the register data, we did not have access to information on many important risk factors for CVEs. We did not have data on lipid profiles, family history of CVD, lifestyle-related risk factors nor on the use of acetylsalicylic acid of the study population.

We reached the same results with the conventional regression model applying standard adjustments as we did with the period-stratified (sequential cohort) model and the cohort-wide PS matched model adjusted for strong predictors of the outcome. Our period-stratified model could probably not discriminate between the characteristics of the atorvastatin and simvastatin initiators and other factors associated with calendar time. Although period was a weak confounder, we did, however, find prescribing dynamics over time as the odds ratios of some of the covariates (number of hospital days, prior CVD, diabetes) varied across the PS models. Furthermore, omitting period from the conventional Cox model did not affect the HR of the outcome. However, a traditional PS matched analysis produced an effect estimate that was the furthest from unity.

In a recent analysis of the effect estimates of cancer chemotherapies that applied a calendar time-specific PS approach [3], a clear confounding effect of the calendar time was found. The definition of the periods was based on drug policy events. Policy events may substantially affect the cost of relatively expensive cancer drugs and thus modify the selection of a therapy. In our study, the length of the accrual periods was based on calendar time, and only one substantial policy change took place during the study period. Although we could identify channeling of the atorvastatin and simvastatin after the policy event, calendar time did not act as a strong confounder in our comparative effectiveness analyses.

This study expands upon the previous work of Rassen et al. [24], who compared the effect estimates for subgroups from the outcome models by applying a PS estimated for a full cohort and also within the subgroups. They observed practically the same effect estimates for the subgroups in the subgroup-specific PS models as in the full cohort PS models. The model for the full cohort PS included interaction terms between the subgroups and strong confounders. When the subgroups were large enough, the validity of the full cohort PS approach was not threatened, and it was more efficient than the stratified analyses. The calendar-time-specific PS matched approach in an analysis of the effectiveness of second-generation antipsychotics on cardiovascular outcomes yielded the same effect estimates as the conventional PS matched approach [4]. Furthermore, preliminary results on the effectiveness of inhaled, long-acting beta-agonists on asthma exacerbations [25] showed that the effect estimates were the same whether or not they were derived from the model using a PS estimated for the entire study period or a PS estimated for specific years and used for matching in the models.

## Conclusions

Clinically, atorvastatin (10 mg) was more effective than simvastatin (20 mg) in preventing CVEs. From the analytical point of view, when accrued data are analyzed, the sequential cohort approach applying PS matching may produce a similar, and as valid, effect estimates as matching for PS within a whole cohort and adjusting the outcome model for strong confounders, at the cost of efficiency. However, without further adjustment, the traditional matching for the cohort-wide PS may lead to less valid estimates. The feasibility of the approach is worth testing in other settings.

## Supporting Information

Table S1 **Characteristics of the initiators of simvastatin and atorvastatin therapy between January 1998 and June 2006 in Finland.**
(PDF)

Table S2 **Number of all initiators of simvastatin and atorvastatin therapy between January 1998 and June 2006 in Finland by 6-month periods, number of those restricted by the first tablet strength (simvastatin, 20 mg, and atorvastatin, 10 mg) and by the start of the follow-up at 270 days since initiation, and those matched by propensity score in each period.**
(PDF)

Table S3 **Covariate balance within selected periods and across all periods, presented as the standardized difference.**
(PDF)

Table S4 **Comparative effectiveness among initiators of atorvastatin (10 mg) versus simvastatin (40 mg) between January 1998 and June 2006 in Finland; hazard ratios for a composite of cardiovascular events^1^ estimated with different Cox proportional hazard regression models.**
(PDF)
