# Correction: Sequential Cohort Design Applying Propensity Score Matching to Analyze the Comparative Effectiveness of Atorvastatin and Simvastatin in Preventing Cardiovascular Events

**DOI:** 10.1371/journal.pone.0101633

**Published:** 2014-07-31

**Authors:** The PLOS ONE Staff

There is an error in Table 1. There are several lines out of place in the original table. Please find a corrected version below.

**Table 1:** Characteristics of the initiators of simvastatin and atorvastatin therapy between January 1998 and June 2006 in Finland. The study population restricted by the strength of the initiating drug and by the start of the follow-up at 270 days since the initiation is presented without and with the sequential matching by propensity score.

	Initiators without matching					Sequentially PS matched				
	Simvastatin 20 mg		Atorvastatin 10 mg			Simvastatin 20 mg		Atorvastatin 10 mg		
	(*n* = 73 868)		(*n* = 96 995)			(*n* = 54 220)		(*n* = 54 220)		
	*n*	(%)	*n*	%	*P* value	*n*	(%)	*n*	%	*P* value
Female	36 612	−49.6	50 107	−51.7	<0.001	27 453	−50.6	27 449	−50.6	*
Age, mean (SD)	60.9	−8	60.4	−8	<0.001	60.7	−8	60.7	−8	*
**Age category**					<0.001					*
45–55 years	21 200	−28.7	30 431	−31.4		15 992	−29.5	15 868	−29.3	
56–65 years	29 252	−39.6	37 674	−38.8		21 400	−39.5	21 338	−39.4	
66–75 years	23 416	−31.7	28 890	−29.8		16 828	−31	17 014	−31.4	
**Number of hospital days during 365 days prior to the initiation**					<0.001					*
0	51 583	−69.8	73 396	−75.7		39 233	−72.4	39 265	−72.4	
1–7	14 202	−19.2	15 908	−16.4		9837	−18.1	9835	−18.1	
8–30	6790	−9.2	6281	−6.5		4262	−7.9	4204	−7.8	
31–365	1293	−1.8	1410	−1.5		888	−1.6	916	−1.7	
**Comorbidities**										
*CVD, PTCA or CAPG in relation to the initiation*					<0.001					*
None in preceding 7 years	63 469	−85.9	82 625	−91.4		48 130	−88.8	48 166	−88.8	
Only earlier than 30 days	3700	−5	4791	−4.9		2846	−5.3	2833	−5.2	
Only during the preceding 30 days	5743	−7.8	2916	−3		2705	−5	2680	−4.9	
Both prior to and during 30 days	956	−1.3	663	−0.7		539	−1	541	−1	
*Hospitalized during 7 years prior to the initiation*										
Diabetes	3589	−4.9	4144	−4.3	<0.001	2471	−4.6	2493	−4.6	*
Hypertension	5269	−7.1	5933	−6.1	<0.001	3593	−6.6	3620	−6.7	*
Stroke	3692	−5	5755	−5.9	<0.001	2972	−5.5	2838	−5.2	0.071
Cardiac insufficiency	990	−1.3	814	−0.8	<0.001	594	−1.1	567	−1.1	*
Atherosclerotic CVD	988	−1.3	1195	−1.2	0.054	710	−1.3	711	−1.3	*
Atherosclerosis in lower legs	416	−0.6	507	−0.5	0.258	295	−0.5	288	−0.5	*
*Hospitalized during 365 days prior to the initiation*										
Atrial fibrillation	957	−1.3	986	−1	<0.001	655	−1.2	614	−1.1	*
Any cancer diagnosis	461	−0.6	578	−0.6	0.458	316	−0.6	315	−0.6	*
COPD/asthma	324	−0.4	291	−0.3	<0.001	207	−0.4	207	−0.4	*
Renal insufficiency	22	0	50	−0.1	0.03	12	0	21	0	*
Dementia	36	−0.1	35	0	0.204	19	0	21	0	*
Psychotic disease	180	−0.2	206	−0.2	0.177	127	−0.2	123	−0.2	*
Depression	189	−0.3	202	−0.2	0.041	124	−0.2	126	−0.2	*
Organ transplantation	8	0	13	0	0.634	3	0	4	0	*
*Comorbidities based on the Special Reimbursement Register* #										
Diabetes	8161	−11.1	10 043	−10.4	<0.001	5703	−10.5	5760	−10.6	*
Hypothyroidism	2494	−3.4	3295	−3.4	0.814	1866	−3.4	1857	−3.4	*
Psychotic disorders	2015	−2.7	2319	−2.4	<0.001	1345	−2.5	1350	−2.5	*
Severe psychotic disease	147	−0.2	137	−0.1	<0.001	96	−0.2	97	−0.2	*
Breast cancer	361	−1	427	−0.9	0.15	256	−1	257	−1	*
Prostate cancer	364	−0.9	387	−0.9	<0.001	243	−0.9	242	−0.9	*
Leukemia	227	−0.3	229	−0.2	0.005	145	−0.3	140	−0.3	*
Gynecologic cancers	42	−0.1	68	−0.1	0.285	34	−0.1	27	−0.1	*
Other cancers	85	−0.1	89	−0.1	0.135	45	−0.1	48	−0.1	*
Prior organ transplantation	69	−0.1	139	−0.1	0.003	56	−0.1	52	−0.1	*
Uremia with dialysis	40	−0.1	104	−0.1	<0.001	28	0	33	−0.1	*
Use of interferon alpha	13	0	19	0	0.766	8	0	9	0	*
Cardiac insufficiency	2001	−2.7	2181	−2.3	<0.001	1341	−2.5	1350	−2.5	*
Rheumatic disease	2117	−2.9	2559	−2.6	<0.001	1492	−2.8	1480	−2.7	*
Asthma	5194	−7	5867	−6.1	<0.001	3576	−6.6	3592	−6.6	*
Chronic hypertension	22 723	−30.8	30 214	−31.1	0.085	16 776	−30.9	16 819	−31	*
CAD	10 917	−14.9	10 434	−10.8	<0.001	7049	−13	7034	−13	*
Dysrhythmia	1707	−2.3	2037	−2.1	<0.001	1231	−2.3	1223	−2.3	*
Familial dyslipidemia	40	−0.1	36	0	0.098	26	−0.1	25	−0.1	*
Dyslipidemia with CAD	3692	−5	2927	−3	<0.001	2256	−4.2	2256	−4.2	*
Clopidogrel use with CAD	74	−0.1	43	0	<0.001	36	−0.1	36	−0.1	*
Clopidogrel use with other indications	235	−0.3	220	−0.2	<0.001	146	−0.3	149	−0.3	*
Use of anti-dementia drugs§	128	−0.2	206	−0.2	0.07	96	−0.2	91	−0.2	*
Parkinsonism	241	−0.3	274	−0.3	0.102	163	−0.3	145	−0.3	*
Epilepsy	847	−1.2	1165	−1.2	0.301	621	−1.2	614	−1.1	*
**Medication**										
*Number* of different preparations purchased during 4 months prior to the initiation					<0.001					
1–5	9571	−13	7626	−7.9		5675	−10.5	5749	−10.6	*
6–10	20 434	−27.7	21 861	−22.5		14 015	−25.9	14 034	−25.9	
11–15	17 596	−23.8	22 806	−23.5		13 108	−24.2	13 087	−24.1	
16–20	11 339	−15.4	17 041	−17.6		8828	−16.3	8884	−16.4	
>20	14 928	−20.2	27 661	−28.5		12 594	−23.2	12 466	−23	
*At least one purchase during 365 days prior to the initiation*										
Diabetes drugs	11 500	−15.6	14 284	−14.7	<0.001	8026	−14.8	8027	−14.8	*
Antithrombotic agents	8608	−11.7	9850	−10.2	<0.001	5887	−10.9	5814	−10.7	*
Organic nitrates and cardiac glycosides	15 772	−21.4	15 877	−16.4	<0.001	10 385	−19.2	10 309	−19	*
Centrally acting hypertension drugs	644	−0.9	746	−0.8	0.019	437	−0.8	441	−0.8	*
Diuretics	11 203	−15.2	14 231	−14.7	<0.001	8066	−14.9	8050	−14.9	*
Peripheral vasodilators	64	−0.1	124	−0.1	0.011	49	−0.1	52	−0.1	*
Beta-blocking agents	31 409	−42.5	36 108	−37.2	<0.001	21 906	−40.4	21 915	−40.4	*
Selective calcium channels blockers	12 678	−17.2	16 133	−16.6	<0.001	9203	−17	9270	−17.1	*
ACEI or ARB	26 292	−36	30 162	−31.1	<0.001	18 166	−33.5	18 176	−33.5	*
Non-statin lipid-lowering drug	544	−0.7	1573	−1.6	<0.001	439	−0.8	472	−0.9	*
Drugs for obstructive airway diseases	7680	−10.4	9293	−9.6	<0.001	5361	−9.9	5387	−9.9	*
Anti-dementia drugs	79	−0.1	111	−0.1	0.645	59	−0.1	54	−0.1	*
Antidepressants	7086	−9.6	9424	−9.7	0.393	5232	−9.7	5172	−9.5	*
Antipsychotics	2088	−2.8	2555	−2.6	0.015	1430	−2.6	1437	−2.7	*
Antineoplastic agents	342	−0.5	521	−0.6	0.032	265	−0.5	256	−0.5	*

CVD, cardiovascular disease; PTCA, percutaneous transluminal coronary angioplasty; CAPG, coronary artery bypass craft surgery; COPD, chronic obstructive pulmonary disease; CAD, coronary artery disease; ACEI, angiotensin–converting-enzyme inhibitor; ABR, angiotensin receptor blocker.

# Eligibility to special reimbursement any time prior to the initiation.

§ Use of donepezil, galantamine, memantine, or rivastigmine.

*P* values are based on Chi^2^ test for categorical variables and Student’s *t* test for continuous variables.

*>0.1.
