# Supplementary material for: Sequential Cohort Design Applying Propensity Score Matching to Analyze the Comparative Effectiveness of Atorvastatin and Simvastatin in Preventing Cardiovascular Events
Source: PLoS One. 2014 Mar 10;9(3):e90325. doi: 10.1371/journal.pone.0090325 (PMC3948677; doi:10.1371/journal.pone.0090325)
Supplement: Table S1 — Characteristics of the initiators of simvastatin and atorvastatin therapy between January 1998 and June 2006 in Finland. (PDF) [file pone.0090325.s001.pdf]

Supporting information

Table S1. Characteristics of the initiators of simvastatin and atorvastatin therapy between January 1998 and June 2006 in Finland.

	Simvastatin (*n*=180 238)		Atorvastatin (*n*=118 623)	
	n	**(%)**	***n***	**(%)**
Female	92 060	(51.1)	58 520	(49.3)
Age in years, mean (SD)	61.4	(8.0)	60.3	(8.0)
**Age category**				
45−55 years	47 259	(26.4)	37 659	(31.6)
56−65 years	70 117	(38.9)	45 974	(38.8)
66−75 years	62 592	(34.7)	34 990	(29.5)
**Number of hospital days during 365 days prior to the initiation**				
0	124 889	(69.3)	86 105	(72.6)
1−7	34 337	(19.1)	21 282	(17.9)
8−30	17 408	(9.7)	9117	(7.7)
31−365	3604	(2.0)	2119	(1.8)
**Comorbidities**				
CVD, PTCA or CAPG in relation to the initiation				
None in preceding 7 years	172 936	(84.9)	105 420	(88.5)
Only earlier than 30 days	11 952	(5.9)	6241	(5.2)
Only during the preceding 30 days	15 548	(7.6)	6276	(5.3)
Both prior to and during 30 days	3171	(1.6)	1214	(1.0)
Hospitalized during 7 years prior to the initiation				
Diabetes	9137	(5.1)	5555	(4.7)
Hypertension	13 256	(7.4)	8328	(7.0)
Stroke	9914	(5.5)	7383	(6.2)
Cardiac insufficiency	2514	(1.4)	1334	(1.1)
Atherosclerotic CVD	2724	(1.5)	1603	(1.4)
Atherosclerosis in lower legs	1157	(0.6)	683	(0.6)
Hospitalized during 365 days prior to the initiation				
Atrial fibrillation	2259	(1.3)	1315	(1.1)
Any cancer diagnosis	1088	(0.6)	754	(0.6)
COPD/Asthma	758	(0.4)	392	(0.3)
Renal insufficiency	80	(0.0)	67	(0.1)
Dementia	115	(0.1)	48	(0.0)
Psychotic disease	418	(0.2)	271	(0.2)
Depression	421	(0.2)	265	(0.2)
Organ transplantation	35	(0.0)	17	(0.0)
Comorbidities based on the Special Reimbursement Register#				
Diabetes	20 610	(11.4)	12 373	(10.4)
Hypothyroidism	6583	(3.7)	3943	(3.3)
Psychotic disorders	4787	(2.7)	2907	(2.5)
Severe psychotic disease	312	(0.2)	177	(0.2)
Breast cancer	8433	(0.9)	513	(0.9)
Prostate cancer	797	(0.9)	501	(0.8)

Continues

Table 1. Continued

	Simvastatin (*n*=180 238)		Atorvastatin (*n*=118 623)	
	n	**(%)**	***n***	**(%)**
Leukemia	474	(0.3)	289	(0.2)
Gynecologic cancers	112	(0.1)	74	(0.1)
Other cancers	202	(0.1)	116	(0.1)
Prior organ transplantation	262	(0.2)	164	(0.1)
Uremia with dialysis	186	(0.1)	129	(0.1)
Use of interferon alpha	29	(0.0)	23	(0.0)
Cardiac insufficiency	5444	(3.0)	2827	(2.4)
Rheumatic disease	5298	(2.9)	3176	(2.7)
Asthma	12 498	(6.9)	7142	(6.0)
Chronic hypertension	57 333	(31.8)	36 491	(30.8)
CAD	30 109	(16.7)	14 923	(12.6)
Dysrhythmia	4299	(2.4)	2558	(2.2)
Familial dyslipidemia	81	(0.0)	89	(0.1)
Dyslipidemia with CAD	8206	(4.6)	4890	(4.1)
Clopidogrel use with CAD	182	(0.1)	110	(0.1)
Clopidogrel use with other indications	507	(0.3)	310	(0.3)
Use of donepezil, galantamine, memantine, or rivastigmine	339	(0.2)	261	(0.2)
Parkinsonism	595	(0.3)	356	(0.3)
Epilepsy	2213	(1.2)	1472	(1.2)
**Medication**				
Number of different reimbursed preparations purchased during 4 months prior to the initiation				
1−5	20 292	(11.3)	9659	(8.1)
6−10	45 668	(25.3)	27 443	(23.1)
11−15	41 668	(23.1)	28 389	(23.9)
16−20	29 127	(16.2)	20 735	(17.5)
>20	43 463	(24.1)	32 406	(27.3)
At least one purchase during 365 days prior to the initiation				
Diabetes drugs	28 263	(15.7)	17 664	(14.9)
Antithrombotic agents	22 368	(12.4)	13 811	(11.5)
Organic nitrates and cardiac glycosides	42 682	(23.7)	22 183	(18.7)
Centrally acting hypertension drugs	1475	(0.8)	949	(0.8)
Diuretics	28 882	(16.0)	17 703	(14.9)
Peripheral vasodilators	217	(0.1)	141	(0.1)
Beta-blocking agents	79 683	(44.2)	46 783	(39.4)
Selective calcium channels blockers	31 943	(17.7)	19 831	(16.7)
ACEI or ARB	61 658	(34.2)	38 362	(32.3)
Non-statin lipid lowering drug	1296	(0.7)	2048	(1.7)
Drugs for obstructive airway diseases	18 648	(10.4)	11 381	(9.6)
Anti-dementia drugs	203	(0.1)	147	(0.1)
Antidepressants	17 056	(9.5)	11 598	(9.8)
Antipsychotics	5062	(2.8)	3175	(2.7)

Continues

Table 1. Continued

	Simvastatin (*n*=180 238)		Atorvastatin (*n*=118 623)	
	n	**(%)**	***n***	**(%)**
Antineoplastic agents	886	(0.5)	627	(0.5)

CVD, cardiovascular disease; PTCA, percutaneous transluminal coronary angioplasty; CAPG, coronary artery bypass craft surgery; COPD, chronic obstructive pulmonary disease; CAD, coronary artery disease; ACEI, angiotensin –converting -enzyme inhibitor; ABR, angiotensin receptor blocker

#Eligibility to special reimbursement any time prior to the initiation
