# Supplementary material for: Sequential Cohort Design Applying Propensity Score Matching to Analyze the Comparative Effectiveness of Atorvastatin and Simvastatin in Preventing Cardiovascular Events
Source: PLoS One. 2014 Mar 10;9(3):e90325. doi: 10.1371/journal.pone.0090325 (PMC3948677; doi:10.1371/journal.pone.0090325)
Supplement: Table S2 — Number of all initiators of simvastatin and atorvastatin therapy between January 1998 and June 2006 in Finland by 6-month periods, number of those restricted by the first tablet strength (simvastatin, 20 mg, and atorvastatin, 10 mg) and by the start of the follow-up at 270 days since initiation, and those matched by propensity score in each period. (PDF) [file pone.0090325.s002.pdf]

Supporting information.

**Table S2.** Number of all initiators of simvastatin and atorvastatin therapy between January 1998 and June 2006 in Finland by 6-month periods, number of those restricted by the first tablet strength (simvastatin, 20 mg, and atorvastatin, 10 mg) and by the start of the follow-up at 270 days since initiation, and those matched by propensity score in each period.

| | Simvastatin | | | Atorvastatin | | |
|---|---|---|---|---|---|---|
| **Period** | **All initiators** ***n*** | **Restricted** ***n*** | **Matched** ***n* (%)** | **All initiators** ***n*** | **Restricted** ***n*** | **Matched** ***n* (%)** |
| 1 | 6296 | 1166 | 882 (75.6) | 1922 | 1747 | 882 (50.5) |
| 2 | 6605 | 1385 | 1206 (87.1) | 3806 | 3434 | 1206 (35.1) |
| 3 | 7978 | 1769 | 1590 (89.9) | 5533 | 4995 | 1590 (31.8) |
| 4 | 8354 | 2152 | 1895 (88.1) | 5517 | 4924 | 1895 (38.5) |
| 5 | 9301 | 2871 | 2463 (85.8) | 7065 | 6286 | 2463 (39.2) |
| 6 | 8486 | 2813 | 2338 (83.1) | 6882 | 5956 | 2338 (39.3) |
| 7 | 8876 | 3148 | 2811 (89.3) | 9381 | 8217 | 2811 (34.2) |
| 8 | 7452 | 2607 | 2382 (91.4) | 8225 | 7232 | 2382 (32.9) |
| 9 | 10 806 | 4415 | 3911 (88.6) | 8751 | 7563 | 3911 (51.7) |
| 10 | 10 576 | 4621 | 3974 (86.0) | 8155 | 6708 | 3974 (59.2) |
| 11 | 11 439 | 5590 | 4890 (87.5) | 8921 | 7195 | 4890 (68.0) |
| 12 | 9287 | 4502 | 3879 (86.2) | 6890 | 5396 | 3879 (71.9) |
| 13 | 10 465 | 5250 | 4744 (90.4) | 8896 | 6632 | 4744 (71.5) |
| 14 | 12 067 | 5838 | 5345 (91.6) | 10281 | 7718 | 5345 (69.3) |
| 15 | 15 950 | 7540 | 5849 (77.6) | 9223 | 6720 | 5849 (87.0) |
| 16 | 15 146 | 7512 | 3831 (51.0) | 5728 | 3987 | 3831 (96.1) |
| 17 | 21 154 | 10 689 | 2230 (20.9) | 3447 | 2285 | 2230 (97.6) |
| All periods | 180 238 | 73 868 | 54 220 (73.4) | 118 623 | 96 995 | 54 220 (55.9) |
