# Supplementary material for: Sequential Cohort Design Applying Propensity Score Matching to Analyze the Comparative Effectiveness of Atorvastatin and Simvastatin in Preventing Cardiovascular Events
Source: PLoS One. 2014 Mar 10;9(3):e90325. doi: 10.1371/journal.pone.0090325 (PMC3948677; doi:10.1371/journal.pone.0090325)
Supplement: Table S3 — Covariate balance within selected periods and across all periods, presented as the standardized difference. (PDF) [file pone.0090325.s003.pdf]

Supporting information.

Table S3. Covariate balance within selected periods and across all periods, presented as the standardized difference.

Covariate	Period[1]	Unmatched cohort	Cohort-wide PS-matched cohort	Sequentially PS-matched cohort
Mean age	1−17	0.061	0.001	0.006
	1	0.006	0.038	0.013
	8	0.043	0.012	0.013
	17	0.057	0.002	0.015
Prior CVD	1−17	0.172	0.007	0.002
	1	0.438	0.210	0.036
	8	0.316	0.118	0.000
	17	0.031	0.175	0.031
Prior stroke	1−17	0.041	0.002	0.011
	1	0.103	0.153	0.023
	8	0.065	0.022	0.024
	17	0.204	0.176	0.000
Diabetes	1−17	0.023	0.001	0.000
	1	0.137	0.201	0.036
	8	0.046	0.064	0.000
	17	0.091	0.080	0.005
Hypertension	1−17	0.008	0.001	0.002
	1	0.060	0.023	0.010
	8	0.025	0.064	0.005
	17	0.067	0.070	0.018

[1]6-month periods starting from January 1998 and ending June 2006

PS = propensity score; CDV = acute myocardial infarction, ischemic cardiac disease, percutaneous coronary intervention, or coronary artery bypass surgery.

Diabetes was based on purchase of reimbursed antidiabetic drugs during the preceding 365 days.
