# Supplementary material for: Sequential Cohort Design Applying Propensity Score Matching to Analyze the Comparative Effectiveness of Atorvastatin and Simvastatin in Preventing Cardiovascular Events
Source: PLoS One. 2014 Mar 10;9(3):e90325. doi: 10.1371/journal.pone.0090325 (PMC3948677; doi:10.1371/journal.pone.0090325)
Supplement: Table S4 — Comparative effectiveness among initiators of atorvastatin (10 mg) versus simvastatin (40 mg) between January 1998 and June 2006 in Finland; hazard ratios for a composite of cardiovascular events1 estimated with different Cox proportional hazard regression models. (PDF) [file pone.0090325.s004.pdf]

Supporting information

Table S4. Comparative effectiveness among initiators of atorvastatin (10 mg) versus simvastatin (40 mg) between January 1998 and June 2006 in Finland; hazard ratios for a composite of cardiovascular events[1] estimated with different Cox proportional hazard regression models

**Models**	**HR (95% CI)**
Conventional outcome models (n=104736)	
Unadjusted	0.53 (0.49-0.57)
Adjusted[2]	0.83 (0.77-0.91)
PS models	
Matched by PS, unadjusted[3] (*n*=14008)	0.94 (0.84-1.06)

HR = hazard ratio; CI = confidence interval; PS = propensity score

[1]Cardiovascular events; hospitalized acute myocardial infarction ischemic cardiac disease (ICD-10 codes I20−I24), percutaneous coronary intervention or coronary artery bypass surgery, and stroke (I63, I64)

[2]Adjusted for all covariates included in the propensity scores, including the period

[3]Period included in the propensity score. Matching within the whole cohort within a 0.01 caliber

[4]Adjusted for the covariates strongly associated with the outcome
